# Evidence for Correlation between Novel Autoantibody against Phospholipid Named Neoself Anti-*β*2-GPI/HLA-DR Antibody and Complement Consumption in Infertile Patients

**DOI:** 10.1155/2023/1318553

**Published:** 2023-09-06

**Authors:** Hirotaka Matsumi

**Affiliations:** MATSUMI Ladies Clinic Mita, Tokyo, Japan

## Abstract

Impaired implantation is one of the causes of infertility. It occurs under vital inflammatory status due to immune hyperactivation. In the innate immune system, the inflammatory response to pathogenic stimuli is initiated by complement activation. Minimal vasculitis associated with complement consumption in infertile patients may be an underlying mechanism for impaired implantation. Antiphospholipid antibodies regulate the inflammatory response. Recently, a novel autoantibody (neoself antibody) against a complex of *β*2-GPI and HLA class II molecules (*β*2-GPI/HLA-DR) has been reported to be an independent autoantibody associated with aPLs. This study investigated the relationship between neoself antibodies and complement consumption in infertile patients with impaired implantation. It was found that decreased C4 levels were strongly related to the increased neoself antibody titers in the serum among those patients whose antibody titers were not as high. On the contrary, serum levels of CH50 and CRP are not correlated with them. These results suggest that neoself antibodies might indicate low-grade inflammation, which causes endometrial vasculitis in impaired implantation of infertile patients.

## 1. Introduction

In 2014, Arase et al. reported that the complexes with misfolding proteins and human leukocyte antigen (HLA) class II molecules transported to the cell surfaces were specific targets for autoantibodies [1]. These are associated with autoimmune diseases, and thus these are named neoself antibodies [2].

Obtained data suggested neoself antigen expression at inflammatory sites in various autoimmune diseases. For example, complexes of IgGH/HLADR and TSHR/HLA-DR are expressed in the synovial membrane in rheumatoid arthritis and the thyroid gland in Graves' disease, respectively.

Among them, anti-*β*2-GPI/HLA-DR antibody (*β*2-GPI neoself antibody) has also been confirmed in antiphospholipid syndrome, which is associated with hyperactivation of immune systems [2]. High concentrations of aPLs, which consist of anti-cardiolipin, anti-*β*2-glycoprotein I, and lupus anticoagulant in the serum, are known to exist in infertile patients [3, 4].

The previous paper from AMED reported that the titers of anti-*β*2-GPI/HLA-DR antibodies are independently high apart from conventional aPLs in infertile women [2].

The etiology of patients with impaired implantation [5] is linked with immune activation and low-grade inflammation. Recent studies reported that complement consumption is progressing in patients with IVF and that anti-cardiolipin and anti-*β*2-glycoprotein I antibodies are associated with adverse IVF outcomes [6, 7].

Recently, anti-*β*2-GPI/HLA-DR antibody has been reported to be expressed in the endometrial tissues and associated with infertility [8].

Therefore, we investigated the relationship between serum levels of complement component 3 (C3), complement component 4 (C4), and total hemolytic complement (CH50) and novel *β*2-GPI neoself antibody. Additionally, serum C-reactive protein (CRP), a marker of inflammation, is also evaluated.

The present study aimed to examine the usefulness of neoself antibodies for detecting complement consumption, which causes low-grade inflammation, one of the reasons for impaired implantation.

## 2. Materials and Methods

### 2.1. Study Design

One hundred two infertile women without tubal and severe male factors at Matsumi Ladies Clinic Mita, Tokyo, Japan, were recruited between 2020 and 2022. The patients had no known history of thrombosis and received no NSAID before recruitment. The population in this study consisted of 43 (42%) infertile patients without a history of miscarriage, regardless of chemical abortion, and 59 (58%) infertile patients with a history of miscarriage.

Serum antibody titers of anti-*β*2-GPI/HLA-DR antibodies were measured at Revorf Co. Ltd. (Tokyo, Japan) in 102 infertile patients. Anti-*β*2-GPI/HLA-DR antibody level was determined as previously described with minor modifications [2]. Informed consent was obtained from all participants, and the Matsumi Ladies Clinic Mita Committees approved the study on Health Research Ethics. The Declaration of Helsinki was followed in all aspects.

### 2.2. Blood Sampling

Venous blood samples were collected from patients by antecubital vein puncture. The first tube was discarded, and blood was sampled in 3.2% sodium citrate tubes. Blood samples were centrifuged at 3000 relative centrifugal force for 30 min at room temperature, and platelet-poor plasma was stored at −20°C until batch analysis. Centrifugation was done within 30 min of the sample collection.

### 2.3. Serum Quantification and Functional Assessment of Complement Proteins

To investigate the activation of the complement consumption, we assessed the concentrations of C3, C4, and CH50 in the serum. CRP was also measured to analyze the inflammation condition.

All patient laboratory analyses were performed by standardized procedures, by the same laboratory technicians, and in the same laboratory facilities. Levels of C3 and C4 were measured using nephelometry with normal ranges of 83–172 mg/dL and 17–51 mg/dL, respectively (Beckman Coulter, California, USA). CH50 was measured using radial immune diffusion with a standard range of 25–48  units/mL (Sanofi Pasteur, Paris, France). These parameters are included in the protocol of all patients evaluated for the first time at the clinical immunology unit. Serum samples were thawed in a water bath at 37°C for 5 min, followed by ultracentrifugation at 17,000 relative centrifugal force for 3 min. The concentrations of complement components were measured in duplicate, and results were obtained from the mean of duplicate measurements, when a coefficient of variation (CV) of samples is below 10%. Samples with a CV above 10% were repeated.

### 2.4. Statistical Analysis

Statistical analyses and figures were performed in JMP. Data distribution was assessed by evaluating Q-Q plots. Data that followed normal distribution are presented as mean ± standard deviation (SD). Data that did not follow a normal distribution are presented as median with interquartile range (IQR). The Pearson correlation coefficient (*r*) is used for evaluating a linear correlation. Unpaired data were compared with an unpaired *t*-test. *P* < 0.05 was considered statistically significant.

## 3. Results

### 3.1. Patient Profile

A total of 102 patients were recruited in this study. Of the total, 43 (42.1%) patients do not have a history of miscarriage, regardless of the presence of chemical abortion. The number of patients with medical records of the absence of miscarriage is 43 (42.1%). The number of patients in the total, according to the history of the number of miscarriages (1, 2, and more than 2), is 15 (14.7%), 30 (29.4%), and 14 (13.7%), respectively.

### 3.2. Distribution of CRP Concentration

**Figure 1:**
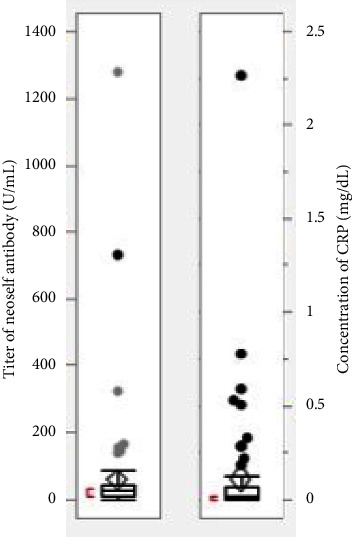
Distribution of CRP concentration and titer of neoself antibody among 102 patients.

The serum levels of CRP were calculated as the marker of inflammation. Among the 102 patients, one had a CRP of 2.26 mg/dl and was considered to have a mild or moderate infection. Of the remaining 101 patients, the levels of CRP are less than 1.0 mg/dl (Figure 1).

### 3.3. Distribution of Neoself Antibodies

The total number of patients was 102. Twelve (11.8%) women were positive for neoself antibodies (cutoff: 73.3 U/ml). As shown in Figure 1, the distribution of titers of neoself antibodies was 10.3 U/ml to 1279.2 U/ml. The minimum value was 10.3 U/ml, and values below that could not be measured. The 97.5% quartile point for these 102 cases was 495.07 U/ml, and the 95% quartile point was 150.05 U/ml.

The top three outliers are 1279.2 U/ml, 729.9 U/ml, and 321.1 U/ml, and each patient profile was summarized in the paragraph titled 4. Excluded Patients.

### 3.4. Recruitment and Statistical Analysis

Among 102 patients with the data of neoself antibody, one with CRP 2.26 mg/dl indicated mild inflammation, and the top three outliers described above are excluded, and the 98 patients are analyzed. Among 98 patients, C3, C4, and CH50 were measured in more than 78 patients, as described below. Similarly, CRP was measured in the 76 patients in the recruited group.

In summary, ninety-eight patients were selected and 58 women were statistically analyzed (Table 1).

### 3.5. The Mean ± SD of Neoself Antibodies of 98 Patients

**Figure 2:**
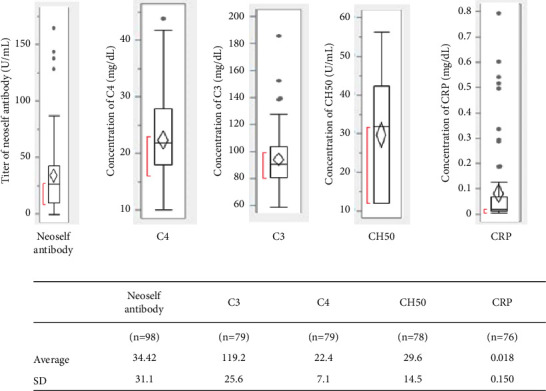
Distribution of C3, C4, and CH50, CRP concentration, and titer of neoself antibody among 98 patients.

Among 98 patients, the mean (±SD) value for the titer of neoself antibody in the serum was 34.2 ± 30.3 (U/ml) (Figure 2).

### 3.6. The Mean ± SD of C3, C4, and CH50 with CRP of 98 Patients

The number of patients with C3, C4, and CH50 data is 79, 79, and 78, respectively. The mean ± SD values for C3, C4, and CH50 were 119.2 ± 25.6 (mg/dL), 22.4 ± 7.1 (mg/dL), and 29.6 ± 14.5 (u/mL), respectively. The number of patients with the data of CRP was 76, and the mean ± SD value for CRP was 0.081 ± 0.150 (mg/dL) (Figure 2).

### 3.7. Correlation between Complement Consumption and Neoself Antibody

The serum levels of CRP >1.0 mg/dL indicate inflammation. The top three outliers for neoself antibodies were excluded from the analysis because these patients have an apparent autoimmune disease and hyperactivity of immune systems.

The details of the four patients consisting of mentioned three and the patient with an elevated serum CRP level are discussed in another section. These four were excluded from the population of 102 patients in the current statistical analysis. In summary, fifty-eight patients were finally recruited and analyzed.

**Figure 3:**
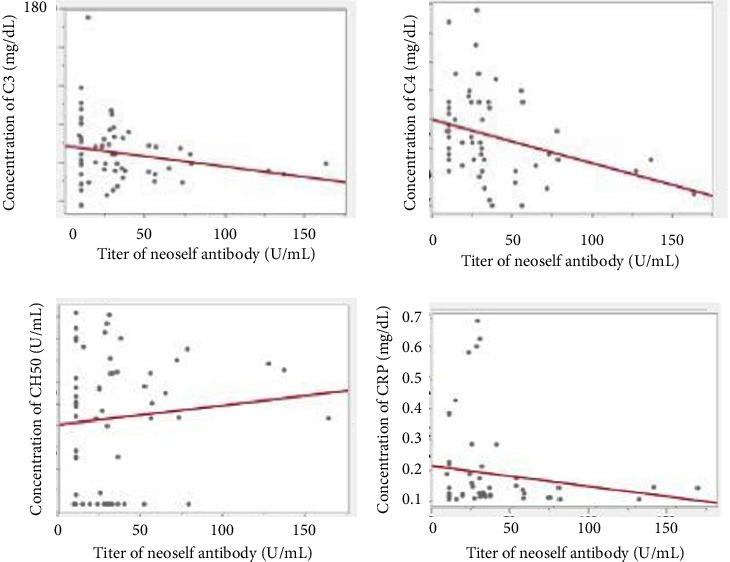
Correlation between neoself antibody and each parameter among 58 finally recruited patients.

Correlations between C3, C4, and CH50 and neoself antibodies were analyzed in the groups in which each complement was measured. The serum levels of C3 decreased with higher titers of neoself antibodies in these recruited patients with no significance (correlation coefficient −0.21, *P* = 0.105). Additionally, C4 was significantly reduced considerably (correlation coefficient −0.33, *P* = 0.0116) (Figure 3). There was no significant difference between CH50 and neoself antibodies (Figure 3).

### 3.8. Correlation between CRP and Neoself Antibody

Similarly, there were no significant differences between CRP and neoself antibodies (Figure 3).

## 4. Excluded Patients

As described above, four patients are excluded from the statistical analysis.

  Case 1 (neoself antibody level = 1279.2 U/ml): a 35-year-old G0 woman had a noticeable increase in autoimmunity with a Th1/2 ratio being 18.1, and conventional antiphospholipid antibodies were positive, with anti-cardiolipin antibody IgG being 15.5 IU/ml.
  Case 2 (321.5 U/ml): A 43-year-old G0 woman with subclinical hypothyroidism and a 2 cm left ovarian, endometrial cyst, medical history of herpes, and drug allergy to LDA. She was diagnosed with chronic endometritis (CD138 positive count 47/20 HPF) and a smoking history of 5 cigarettes/per day for 20 years.
  Case 3 (729.9U /ml): A 28-year-old G0 woman with a history of 4 chemical abortions. She had chronic lymphocytic thyroiditis (Hashimoto's disease) and endometrial polyps with chronic endometriosis. The titers of antibodies for thyroid are anti-TPO antibody 1060 U/ml and anti-thyroglobulin antibody 6470 U/ml. The immune system was hyperactive, with NK cell activity being 50 U/ml.
  Case 4 (CRP = 2.26 mg/ml): A 33-year-old G0 woman with painless thyroiditis with oral intake of 25 ug of levothyroxine. Hysteroscopy revealed multiple endometrial polyps. She underwent endometrial polyp treatment, consisting of hysteroscopic polypectomy and oral intake of doxycycline for chronic endometritis. The neoself antibody titer six months after treatment was 33.1 U/ml, within normal limits. The serum level of CRP was 2.26 mg/ml. High levels of D-dimer and PT fragment F1 + 2 in the plasma indicated that her coagulation ability was activated.

## 5. Discussion

Impaired implantation is one of the most crucial causes of infertility. Subclinical vasculitis, undetectable in the thrombophilic phenotype, is one of the underlying mechanisms of this disease. The phenotype of this early vasculitis is related to an increase in complement consumption without an elevated CRP in the serum.

The complementary system is composed of several small proteins. Three biochemical pathways activate the complement system: the classical complement pathway, the alternative complement pathway, and the lectin pathway. C3 and C4 are markers of the alternative complement pathway and the lectin pathway, respectively. CH50 is a test that collectively measures the activity of the classical complement pathway.

Serum CRP levels below 1.0 mg/dl represent chronic or mild inflammation, while levels above 1.0 mg/dl represent moderate or higher inflammation due to acute infection or other factors.

In the present study, one patient with acute infection was excluded from the recruited 102 patients by setting the cutoff value at 1.0 mg/dl because she has different pathophysiology. Among the 102 patients, three outliers were in the top three patients of neoself antibody titers. These patients had predisposing factors, such as the hyperactivated condition of the immune systems and solid autoimmune abnormalities, which will be discussed below. The titers of neoself antibody in three cases (3/102) were greater than 200 U/ml.

As shown in Table 2, these three patients were excluded from the recruited patients because of the presence of autoimmune abnormalities such as hypothyroidism due to autoantibodies, hyperactivity of NK cells, and abnormal Th1/Th2 balance, which were considered to complicate another pathological condition.

Four patients were excluded from the 102 recruited patients for mathematically and medically valid reasons, and statistical analyses were performed for the remaining 98 patients.

Fifty-eight among 98 patients were finally analyzed concerning the correlation between neoself antibodies and inflammatory biomarkers.

When examined in these 98 patients with less than 200 U/ml, the incidence of low serum C4 levels was significantly correlated with elevated serum titers of neoself antibodies. Similarly, serum C3 was associated with elevated serum titers of neoself antibodies without significance.

There was no significant correlation between neoself antibody titers and CH50 or CRP levels in the serum.

A previous study revealed that elevated serum IgA subclass anti-*β*2-GPI antibodies and immune complexes of *β*2-GPI strongly correlated with decreased serum C3 and C4 levels in infertile patients [9, 10]. Another study found that serum levels of C3 and C4 were higher in patients who had experienced two previous miscarriages and delivered subsequent children than in women who had experienced a third miscarriage [11].

In other words, the higher the low-grade inflammation, the higher the complement consumption, and the lower the serum complement titer is, the more likely a patient with repeated miscarriages will have an additional third miscarriage.

In our study, C4 levels were significantly lower with increasing antibody titers in the serum when titers of neoself antibodies were less than 200 U/ml.

Our findings were consistent with the results of the two studies mentioned above. In the infertile patients, the elevated neoself antibody titers were suggested to signify increased complement consumption associated with low-grade inflammation. In other words, neoself antibodies can be proposed as a new biomarker to detect a systemic chronic inflammatory state that leads to vasculitis of endometrial tissue in infertile patients with impaired implantation.

Several antibodies against phospholipids, such as LA, anti-CL, and anti-*β*2-GPI antibodies (IgG and IgM), are associated with immune abnormalities, vascular inflammation, and subsequent thrombosis. The coagulation system is a partner of the inflammatory response. The cascade of the coagulation system is initiated by the same stimuli that trigger the inflammatory response.

None of our recruited 102 patients had apparent thrombosis in the medical record. However, we speculate that neoself antibodies can depict early clinically asymptomatic minimal vasculitis in the preliminary stages of symptomatic thrombosis. Therefore, patients who are positive for neoself antibodies may benefit from treatment with oral intake of low-dose aspirin, which reduces inflammation and the resulting thrombosis.

The AMED team reports that approximately 20% of repeated pregnancy loss patients are neoself antibody positive. Repeated pregnancy loss is often associated with thrombosis, which occurs as a result of moderate or severe vasculitis. The majority of the patients in our study are undiagnosed with repeated pregnancy loss but diagnosed with impaired implantation, one of the reasons for infertility. In other words, the target of our study patients does not have moderate systemic vasculitis. They are under the condition of early vasculitis of the endometrium. In addition, thrombosis, one of the clinical manifestations of repeated pregnancy loss, was not predominantly noticeable in the patient background as far as the medical history was concerned. Thus, about 12% of those patients would have been positive for neoself antibodies.

Neoself antibodies are associated with chronic low-grade inflammation in infertile patients, some of which result in repeated pregnancy loss. Almost no patients were positive for neoself antibodies but positive for conventional antiphospholipid antibodies (data not shown). Some patients with positive serum aPLs are asymptomatic, with no thrombosis. The results indicate that the titer of the neoself antibody detects the condition of complement consumption more sensitively than the conventional aPLs [12, 13].

Moreover, although the cutoff value of neoself antibodies is 73.3 U/ml, titers below the cutoff value are medically significant in our study. They may reflect a decrease in complement, i.e., inflammatory changes involving early endometrial vasculature brought about by systemic chronic low-grade inflammation, which results in impaired implantation.

## 6. Conclusions

The present study investigated the relationship between neoself antibodies and complement components and CRP, indicators of inflammatory status, in infertile patients with impaired implantation. The decreased serum C4 levels were strongly related to the increased neoself antibody titers.

On the contrary, serum levels of CH50 and CRP are not correlated.

Serum complement consumption is associated with the hyperactivated status of immune systems. Almost all the patients are negative for conventional aPLs. Thus, neoself antibodies may be more beneficial than traditional antiphospholipid antibodies for hyperactivated immune status in infertile patients with impaired implantation.

Early screening for neoself antibodies in infertile patients should be considered for couples undergoing general infertility treatment before IVF. The number of patients enrolled in this study is small. A more accurate analysis of a more significant number of patients is expected.

## Figures and Tables

**Table 1:** Schematic diagram for patient recruitment and study design: recruitment of 58 patients from 102 patients.

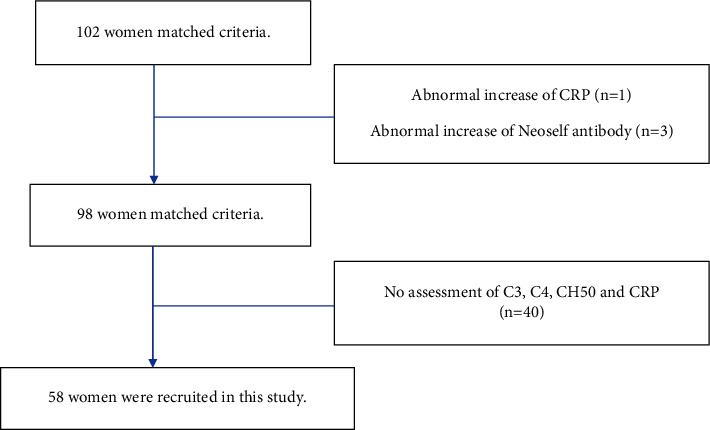

**Table 2:** Excluded cases.

	Age (year)	Neoself antibody (U/mL)	C3 (mg/dL)	C4 (mg/dL)	CH50 (U/mL)	CRP (mg/dL)	Comments
Case 1	35	1279.2	99	22	45.8	0.038	Autoimmunity (Th1/2 ratio: 18.1) Conventional aPLs positive (anti-cardiolipin IgG: 15.5 IU/ml)

Case 2	43	321.5	99	16	34.4	0.007	Medical history of herpes Smoking history of 5 cigarettes/per day for 20 years Drug allergy to LDA Subclinical hypothyroidism, left endometrial ovarian cyst Chronic endometritis

Case 3	28	729.9	165	27	44.5	0.08	History of 4 times chemical abortions Chronic lymphocytic thyroiditis (Hashimoto's disease) Endometrial polyps with chronic endometriosis Hyperimmunoactivity (NK cell activity: 50 U/ml)

Case 4	33	33.1	131	32	50.8	2.26	Hypercoagulated status, painless thyroiditis Multiple endometrial polyps

## Data Availability

The data used to support the findings of this study are available from the corresponding author upon request.
